# Behavioral responses of free-flying *Drosophila melanogaster* to shiny, reflecting surfaces

**DOI:** 10.1007/s00359-023-01676-0

**Published:** 2023-10-05

**Authors:** Thomas F. Mathejczyk, Édouard J. Babo, Erik Schönlein, Nikolai V. Grinda, Andreas Greiner, Nina Okrožnik, Gregor Belušič, Mathias F. Wernet

**Affiliations:** 1 Division of Neurobiology, Institute of Biology, Fachbereich Biologie, Chemie and Pharmazie, Freie Universität Berlin, Königin-Luise Strasse 1-3, 14195 Berlin, Germany; 2 Department of Biology, Biotechnical Faculty, University of Ljubljana, Ljubljana, Slovenia

**Keywords:** *Drosophila*, Polarization vision, Behavior, Thirst

## Abstract

Active locomotion plays an important role in the life of many animals, permitting them to explore the environment, find vital resources, and escape predators. Most insect species rely on a combination of visual cues such as celestial bodies, landmarks, or linearly polarized light to navigate or orient themselves in their surroundings. In nature, linearly polarized light can arise either from atmospheric scattering or from reflections off shiny non-metallic surfaces like water. Multiple reports have described different behavioral responses of various insects to such shiny surfaces. Our goal was to test whether free-flying *Drosophila melanogaster*, a molecular genetic model organism and behavioral generalist, also manifests specific behavioral responses when confronted with such polarized reflections. Fruit flies were placed in a custom-built arena with controlled environmental parameters (temperature, humidity, and light intensity). Flight detections and landings were quantified for three different stimuli: a diffusely reflecting matt plate, a small patch of shiny acetate film, and real water. We compared hydrated and dehydrated fly populations, since the state of hydration may change the motivation of flies to seek or avoid water. Our analysis reveals for the first time that flying fruit flies indeed use vision to avoid flying over shiny surfaces.

**Supplementary Information:**

The online version contains supplementary material available at 10.1007/s00359-023-01676-0.

## Introduction

The ability to use visual cues for improving their navigation is one of the most prominent locomotive achievements of insects (Heinze 2017). This requires animals to perceive their position within their habitat and/or to integrate this signal with spatial memory in the case of returning to their previous location (Collett et al. 2013; Heinze 2017). To navigate or orient, insects combine diverse sensory cues which provide them with information about their surroundings, and consequently induce an adaptive behavior such as following a trajectory (Heinze 2017). Many species have evolved integrated navigational mechanisms, relying on global signals in the sky and/or local signals like landmarks (Collett et al. 2014; El Jundi et al. 2016), wind direction (Dacke et al. 2019), and idiothetic signals like optic flow (Mauss and Borst 2020) and proprioception (Wittlinger et al. 2006). The celestial pattern of linearly polarized skylight is used by many insect species for improving their orientation or navigation skills (Heinze 2017; Wehner and Labhart 2006). The different angles of polarization of skylight, scattered in the atmosphere, create a polarization pattern across the sky which changes according to the location of the sun (Wehner 2001; Heinze 2017; Mathejczyk and Wernet 2017). Many insects have evolved specialized visual systems, including specialized retinal detectors as well as underlying circuitry, to use this pattern for navigation and orientation (Labhart and Meyer 1999; Homberg 2015; Dacke and El Jundi 2018; Sancer et al. 2019, 2020; Kind et al. 2020). Importantly, sunlight also becomes linearly polarized through reflections off shiny surfaces like water, animal or plant cuticles, or any non-metallic shiny surfaces (Wehner 2001). Upon reflection from a flat surface, the angle of linear polarization becomes horizontally polarized with reflected light reaching a maximum degree of linear polarization (DoLP) at the Brewster’s angle (53° from normal for air–water interface, see Wehner 2001). The DoLP is a measure for the portion of light being polarized, ranging from 0% (unpolarized) to 100% (fully polarized).

Reflected polarized light can provide crucial and reliable detail for the detection of a variety of salient stimuli, like water bodies, shiny food, insect cuticles, or even an appropriate place for oviposition (Mathejczyk and Wernet 2017; Heinloth et al. 2018; Yadav and Shein-Idelson 2021). Such ventral detection of polarized light has been demonstrated in a variety of flying insects, ranging from locusts (Shashar et al. 2005) and hemipterans (Schwind 1984), to dragonflies (Wildermuth 1998) and butterflies (Stewart et al 2019; Kelber 1999), chironomids (Lerner et al. 2008), and mayflies (Farkas et al. 2016). Furthermore, it has been shown that, equivalent to those evoked by water, shiny non-metallic surfaces evoke behavioral responses in semi-aquatic insects (Kriska et al. 2007; Farkas et al. 2016). The behavioral response to polarized reflections is attractive in the case of the backswimmer *Notonecta glauca,* which manifests a diving reflex when presented to a polarized surface (Schwind 1984, 1985) or repulsive in the case of the desert locust *Schistocerca gregaria*, which avoids linearly polarized surfaces, presumably to avoid drowning (Shashar et al. 2005). The retinal detectors of horizontally polarized reflections remain largely unclear, with only very few exceptions where photoreceptors with untwisted, orthogonal microvilli (an ultrastructural hallmark of polarization sensitivity) were described in the ventral eye (Schneider and Langer 1969; Schwind 1983; Meglic et al. 2019).

The *Drosophila melanogaster* retina is composed of approximately 800 ommatidia each containing 8 light-sensitive photoreceptors (named R1–R8). Six outer photoreceptors (R1–R6) surround the inner photoreceptors R7 and R8 (Kind et al. 2021). Based on the molecular and physiological characteristics of inner photoreceptors, ommatidia can be divided into at least three groups, called pale, yellow, and DRA (Wernet et al. 2015; Kind et al. 2021). Together, pale and yellow ommatidia form the genetically specified color vision detection system of the fly retina and are randomly distributed at an uneven ratio with 30% pale and 70% yellow (Feiler et al. 1992; Wernet et al. 2006; Bell et al. 2007). Only along the dorsal edge of the fly eye, in the so-called dorsal rim area (DRA), inner photoreceptors R7 and R8 from the same ommatidia form homochromatic polarization sensors with orthogonally arranged microvilli (Wada 1974a, b; Fortini and Rubin 1990; Wernet et al. 2003). It has been shown that the DRA ommatidia of *Drosophila* are necessary and sufficient to detect linearly polarized skylight (Wernet et al. 2012). Surprisingly, *Drosophila* can also perceive linearly polarized light with the ventral half of its eye, despite the lack of DRA-like ommatidia there (Wolf et al. 1980; Wernet et al. 2012; Velez et al. 2014a, b).

Although polarotaxis in *Drosophila* has been well studied in tethered flight for both dorsally and, in some cases, ventrally presented stimuli (Wolf et al. 1980; Weir and Dickinson 2012; Velez et al. 2014a, b; Warren et al. 2018; Mathejczyk and Wernet 2019, 2020), no experiments using free-flying fruit flies have yet been conducted. The aim of this study was, therefore, to determine whether freely flying flies would interact with potentially linearly polarizing surfaces, by analyzing both flight trajectories as well as the distribution of landings. *Drosophila melanogaster* flying freely inside a bright, white arena were presented either one of three different visual stimuli: (i) only a weakly polarizing white matt acrylic base plate, (ii) an additional small piece of shiny, clear acetate film placed in its center, or (iii) a small patch of water. The weakly polarized matt acrylic plate served as the negative, unpolarized control. The round piece of clear acetate film was used to induce polarized reflections without any olfactory water cue, while keeping other visual cues like the intensity of reflected light mostly unaffected. The water body was designed to only slightly alter the visual appearance of the white acrylic plate, while providing an olfactory cue. Finally, since *Drosophila* can be both repelled by, and attracted to water, depending on their physiological state (Ji and Zhu 2015), we decided to test both hydrated and dehydrated populations.

## Materials and methods

### Flies

All experiments were conducted using 4-day-old male and female *Drosophila melanogaster* (strain Top Banana). Flies were raised on standard medium at constant temperature (25 °C) and humidity (50–60%) within a 12/12 h light/dark cycle. For desiccation, flies were transferred into vials containing desiccant (Drierite^®^) at the bottom (about 1 cm fill height) covered by a small ball of cotton wool to avoid any contact of the flies with the desiccant. Flies were desiccated for 6 h with the desiccation vials placed in the incubator (25 °C, 50–60% humidity). All experiments involving hydrated flies were performed up to 3 h after their subjective “sunrise” (morning activity peak). To allow for 6 h desiccation, all experiments involving dehydrated flies were done approximately 2 h before their subjective “sunset” (evening activity peak).

### Experimental setup

To test behavioral responses toward shiny/linearly polarizing surfaces during flight, we designed and constructed a custom behavioral assay (Supplementary Figure S1). In this assay, flies were transferred into a white plastic bucket (diameter approximately 30 cm, height 30 cm) with the base cut off and filmed (Point Grey^®^ Blackfly BFLY-PGE-2356 M + Lee Filters 87C infrared pass filter) from above using near-infrared illumination (LED array, 850 nm) coming from below. The focus of the camera was set to the bottom of the arena. All surfaces visible to the flies within the setup (except the camera lens) were made out of white materials to reduce unwanted intensity artifacts. White cloth was attached to the top of the arena to prevent flies from escaping during experiments. Visible (white light) illumination was provided using RGB LED strips (12 V at 0.1A total) at the top of the arena for which the red, green, and blue channels were calibrated iso-quantally using a photo-spectrometer (Ocean Optics Flame UV–VIS). We did not include UV LEDs since we had previously shown that behavioral responses of walking *Drosophila* populations to polarized light perceived with the ventral half of the eye are efficiently driven by longer wavelength (green light), or when outer photoreceptors R1–6 were the only ones active (rh1-norpA rescues) (Wernet et al. 2012). A matt (surface manually roughed up using sandpaper) white acrylic plate (320 mm × 320 mm, 3 mm thick) served as mostly unpolarizing arena bottom for visual light coming from above, as well as a diffuser for the near-infrared illumination coming from below. The apparatus was placed within a black metal enclosure for optical shielding and climate control. The temperature was upregulated using a carbon heater (#100575, termowelt.de) placed at the bottom of the metal enclosure and an external PID controller (RT4-121-Tr21Sd, pohltechnic.com). We constructed two small humidifiers by placing small fans (40 mm × 40 mm) on top of cut plastic bottles with ventilation holes (filled with water and pieces of replacement humidifier filter Philips 2000 HU4801/02). The humidifiers were controlled using an external hygrostat.

### Visual stimuli

We tested three different visual stimuli presented at the bottom of the arena: an all-matt white acrylic plate showing an overall low degree of linear polarization (DoLP) of reflected light, a matt white acrylic plate with a shiny, highly reflective center (60 mm diameter transparent acetate film, 1CLASSIC 6070), and a matt white acrylic plate with a 60 mm diameter, 1 mm deep (sloped walls) indentation in the center, filled with water (Supplementary Figure S1c–e).

### Polarimetry

Polarimetric and intensity measurements were acquired using a polarimetry camera (PHX050S1-PC, Lucid Vision Labs). To image the center of the arena from different vertical (elevation) angles, a tripod in combination with an electronic level was used. For these measurements, we also replaced the white bucket with vertically cut-in-half bucket of the same type. This allowed for imaging the arena center from a variety of angles (15°–60° elevation), as well as capturing reflections from the bucket walls, as would also be present during experiments. Polarimetric and intensity images were saved as 8-bit greyscale TIFF files, and the mean DoLP and intensity for zone 1 and 2 were quantified using FIJI (Schindelin et al. 2012).

### Experimental procedure and data acquisition

All experiments were conducted at 30 °C and 50–60% relative humidity. Before each experiment, approximately 100 flies were transferred into empty vials and counted manually. Flies were introduced to the arena by inserting the vial through a slit between the white cloth and the bucket and letting flies enter on their own. Each experiment was recorded for 60 min (43fps, 8-bit greyscale, 580 × 580 pixel resolution) and videos were saved using MJpeg compression. Each experimental condition was recorded at least eight times with different batches of flies and after each recording, the flies were vacuumed out of the arena.

### Tracking of flight behavior

To separate flying from walking flies and to track their positional data over time, we developed a custom Matlab (MathWorks) script. From the recorded videos, the code computes intensity differences between consecutive frames for each pixel. At the framerate at which the videos were recorded (43 fps), intensity differences between consecutive frames for walking flies were very low due to their relatively slow walking speed. However, due to the higher flight velocities, flying flies usually moved over a larger distance than their body length between consecutive frames, resulting in large intensity differences at the position where a flying fly ‘appears’ in a consecutive frame. These intensity differences were filtered, thresholded and ultimately used to locate the X and Y coordinates of flight occurrences for many flies simultaneously. This method allows for quick quantification of probabilistic distributions of flight occurrences in large fly populations without having to keep the tracking identities of individual flies for discriminating between walking and flight. To verify the robustness of this method, 8 h of videos containing the thresholded files were compared frame by frame to the original recordings. Depending on the flight angle, in some cases (e.g., a fly flying directly towards the camera), flight activity could not be detected as such due to low intensity differences between consecutive frames, resulting in slight under-sampling. However, due to the large number of flies and observations, overall flight activity is represented very robustly using this method. Since the focus of the camera was set to the bottom of the arena, siting or walking flies appeared out of focus and were subsequently not detected by the tracking code. Therefore, tracking included only flies flying at a certain distance from the camera lens.

### Tracking of landings

Landings were tracked manually using FIJI (Schindelin et al 2012). For tracking, every ten consecutive frames of the original video were converted into a minimal intensity projection, allowing for better visibility of flight trajectories and also an increased tracking speed. Whenever a landing was observed, a point marker was placed, saving the X and Y coordinates of each landing. Due to the very high number of landings of dehydrated flies, for matt dehydrated and shiny film dehydrated recordings, we only tracked the first 1000 landings per video and normalized this data for the shorter sample time.

### Statistical analysis

Statistical analysis was performed using Matlab qq (MathWorks). To calculate the distribution probability for flight and landing detections, we divided the arena into five concentric zones, with the center zone having a diameter of 60 mm to represent the shiny acetate film and water stimuli and each consecutive outer zone having a 60 mm larger diameter than the next smaller area. Flight detections were defined as the number of thresholded (flying) flies per frame. The number of flight detections and landings were calculated for each zone and normalized for the area of the respective zones to finally calculate the normalized detection probability in %. Normalized detection probabilities between zone 1 and zone 2 were compared using the two-sample *t *test for flights and landings, respectively. For flight and landing statistics, we counted 1 h of video recording as one sample (*n* = 1). The number of tested flies and detections for each recorded video and condition is described in detail in supplementary table 1.

## Results

### Behavioral setup and stimulus characterization

**Fig. 1:**
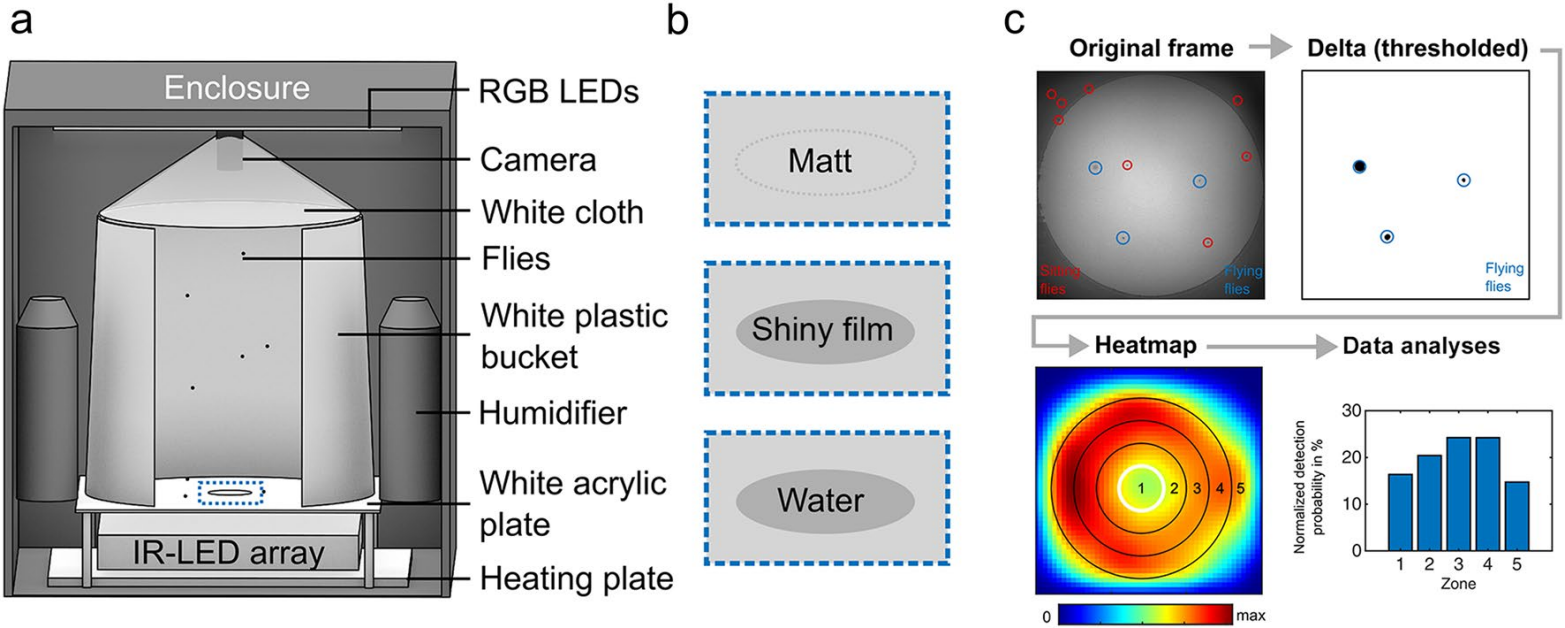
Data acquisition and processing. **a** Schematic of the flight arena setup with all main components (see material and methods). **b** Enlarged sections of the arena center in (**a**) summarizing the three bottom-plate stimuli (see material and methods). **c** Flow chart illustrating the procedure for detecting flying flies in recorded videos and analyzing the spatial distribution of flight detections. The heat map shows the spatial distribution of flight detections of an exemplary 1 h video recording

Here we present a novel assay allowing for probabilistic quantifications of spatial flight distributions (Fig. 1, Supplementary Fig. S1). Freely moving populations of wild-type flies were filmed from above against a near-infrared illumination within a temperature- and humidity-controlled cylindrical arena (Fig. 1a). To test the flies’ behavioral responses toward either one of three different stimuli, the bottom of the arena was either completely matt (rough surface), or equipped either with an additional small piece of shiny acetate film in the center, or with a small body of water at the same location (Fig. 1b). We also developed a computationally fast and easy-to-use procedure of automatically distinguishing flying from walking flies based on intensity differences of consecutive video frames, allowing for the quantification of probabilistic spatial distributions during flight (Fig. 1c) (see Materials and methods).

**Fig. 2:**
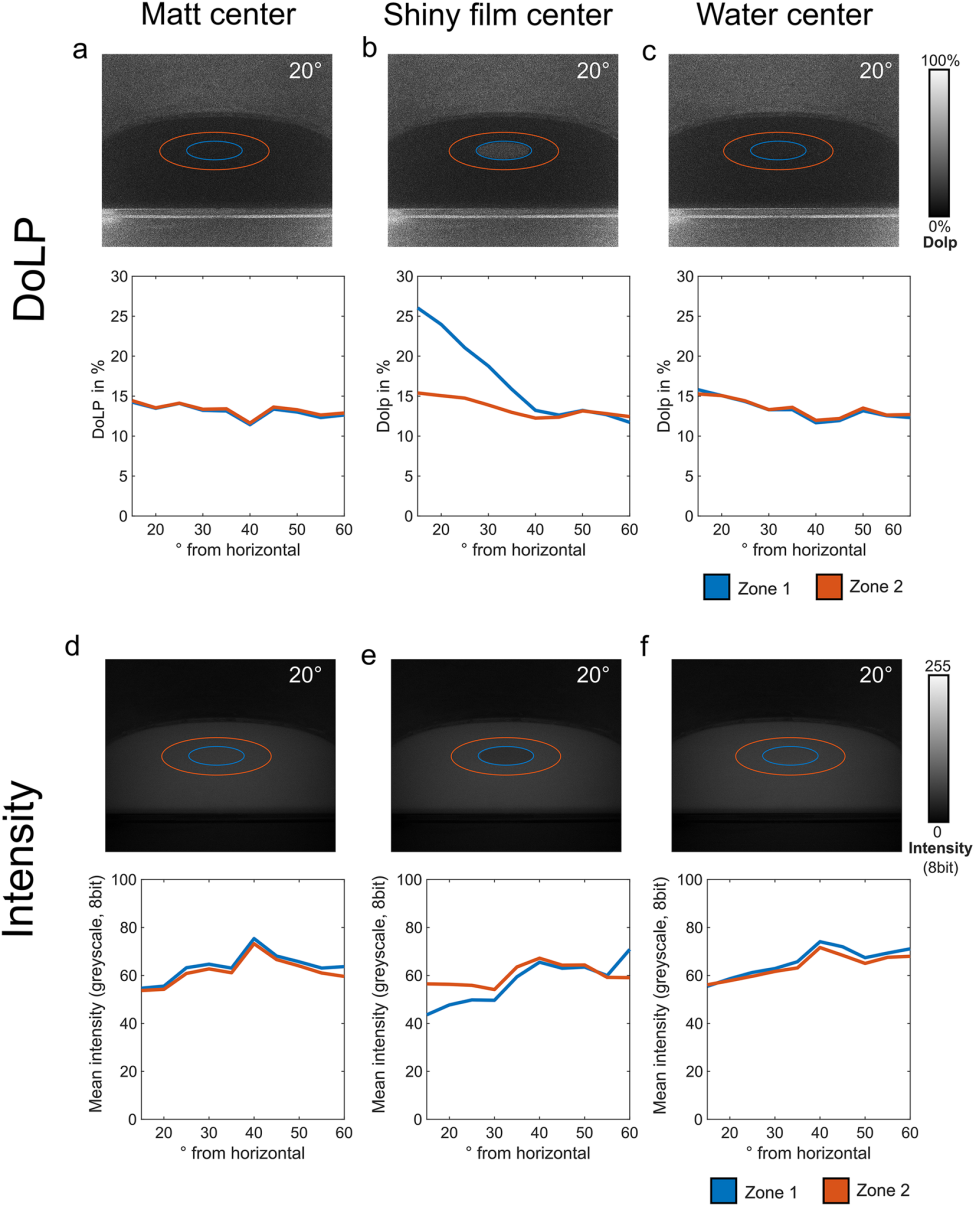
Polarimetric characterization of the stimuli used. **a–c** Polarimetric quantification of the degree of linear polarization (DoLP) of the center (zone 1) vs. immediate surround (zone 2) for the three stimulus conditions used across different incident angles (see material and methods). **e–f** Quantification of main intensity of the center (zone 1) vs. immediate surround (zone 2) for the three stimulus conditions used across different incident angles

Polarimetric characterization of the three different visual stimulus conditions used here was performed as previously described (Meglic et al. 2019), and revealed relatively low values for the degree of linear polarization (DoLP) of around 15%, across all tested elevation angles for both the matt and water stimulus condition and also for the matt region surrounding the shiny acetate film (zone 2), across all stimulus conditions (Fig. 2a, b, c). As expected, an elevated degree of linear polarization of around 25% was measured only when the shiny acetate film was placed in the center of the matt plate (Fig. 2b). Simultaneously performed intensity measurements revealed only little intensity differences between the centrally located zone 1 and the surrounding zone 2 for the matt and water stimulus condition whereas the intensity of the shiny acetate film center was even lower than its’ surrounding zone 2 at low elevation angles and slightly higher only at 60° elevation (Fig. 2d–e). Hence, a shiny acetate film placed at the center of the arena was from most fly aspects not significantly brighter than the surrounding matt white surface.

### Flight behavior

**Fig. 3:**
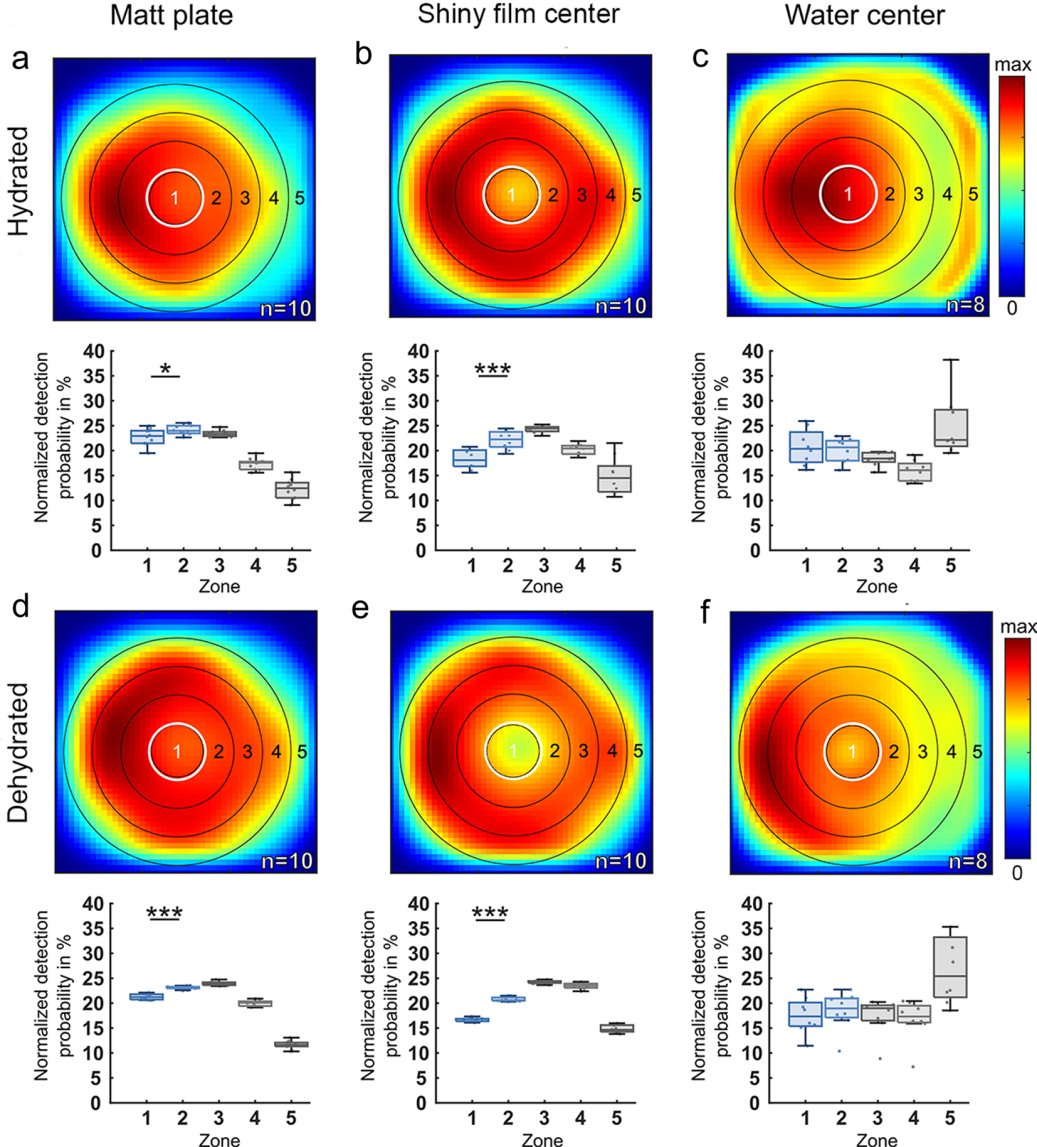
Spatial distributions of flight detections of hydrated vs. dehydrated flies in response to different stimuli. **a**–**c** Summary and direct comparison of the spatial distribution of flight detections of hydrated flies vs. **d**–**f** dehydrated flies in the arena after 60 min of recording of three experimental conditions: (**a**), (**d**) completely matt surface; (**b**), (**e**) matt plate with a shiny acetate film center; (**c**), (**f**) matt plate with a water center. A normalized heat map of the spatial distribution of flight detections is shown, as well as the normalized detection probability for flying *Drosophila* to be in each concentric zone. n indicates the number of 60-min recordings. Differences between zone 1 and zone 2: *p < 0.05, **p < 0.01, ***p < 0.001

**Fig. 4:**
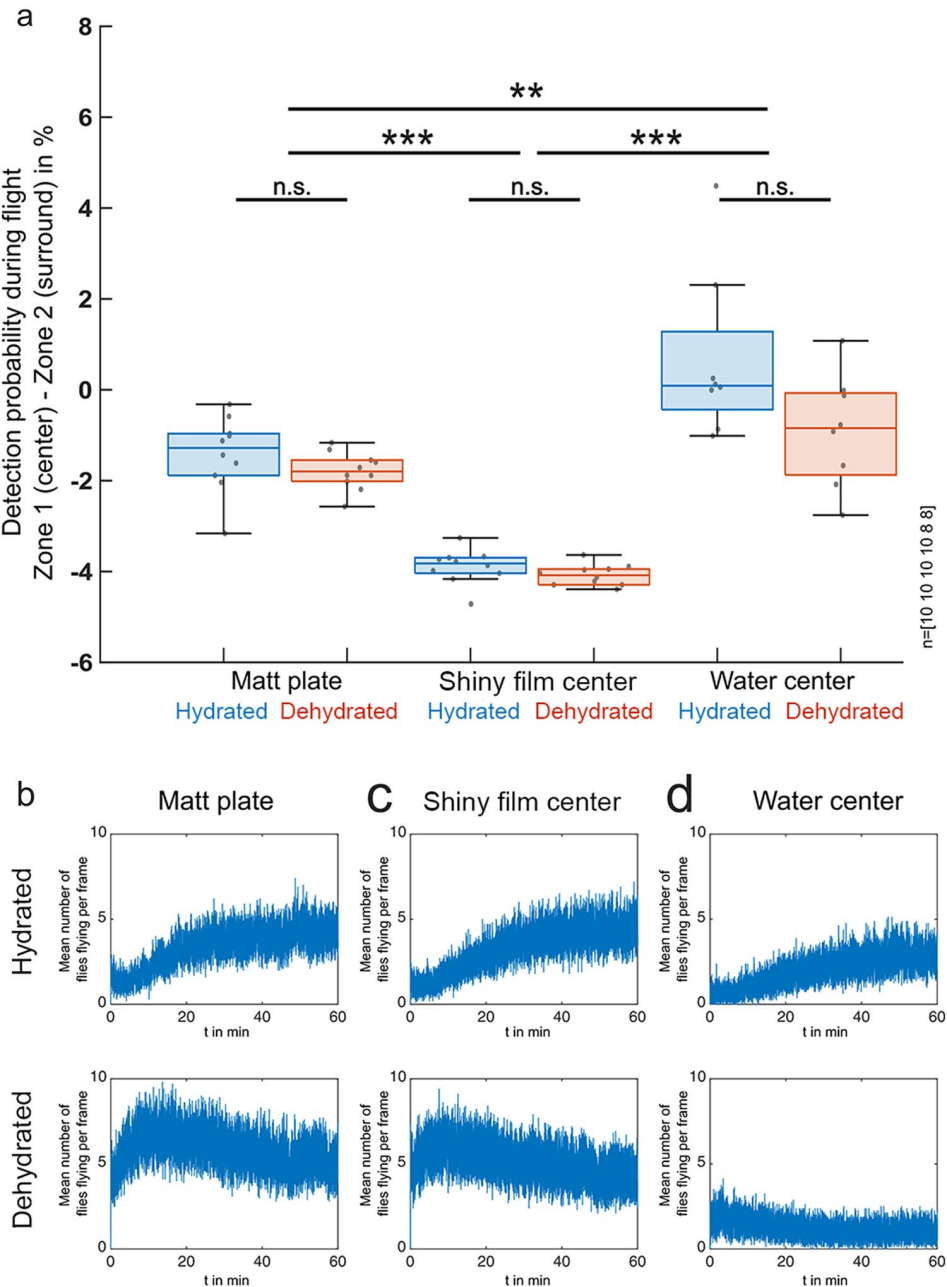
Statistics of hydrated vs. dehydrated flies and overall flight activity. **a** Statistical summary of detection probability during flight (zone 1–zone 2), directly comparing hydrated and dehydrated flies in all three experimental conditions. Number of 60-min recordings from left to right indicated at the right. *p < 0.05, **p < 0.01, ***p < 0.001. **b**–**d** Summary and direct comparison of flight activity (mean number of flies flying per frame) for hydrated flies (top) vs. dehydrated flies (bottom) in the arena after 60 min of recording of three experimental conditions: (**b**) completely matt surface; **c** matt plate with a shiny acetate film in the center; (**d**) matt plate with a water center

For the matt stimulus, we found only small but significant differences in flight detection probability between zone 1 (center) and zone 2 (surround) in dehydrated (*p* < 0.001) as well as in hydrated (*p* < 0.05) fly populations (Fig. 3a, d). For the water stimulus, we did not find significant differences in flight detection probability between zone 1 and zone 2 in dehydrated and hydrated fly populations (Fig. 3c, f). In contrast, the same analysis revealed a strongly reduced flight detection probability above zone 1 when the shiny acetate film was placed there, as compared to its surrounding matt zone 2 (Fig. 3b). This effect was even more pronounced in dehydrated flies (Fig. 3e). Differences in flight detection probability between zone 1 and zone 2 were significantly larger with the shiny acetate film in the center when directly compared to the matt and water stimulus, respectively (Fig. 4a), but did not differ significantly between dehydrated and hydrated flies. We observed a tendency of flies to predominantly accumulate on the left side of the arena. Since we covered the setup with blackout cloth for every experiment to block off external stray light, it is likely that this uneven distribution is an artifact of the air conditioner in the room creating a temperature gradient of up to 1 °C within the setup.

Calculating the average number of flying flies per frame over time allowed us to also quantify flight activity dynamics for both dehydrated and hydrated flies, under all stimulus conditions. When presented with a matt or shiny center, respectively, average flight activity increased over time in hydrated flies, whereas dehydrated flies showed high flight activity from the beginning of the experiment (Fig. 4b–d). However, when presented with water in the center, both dehydrated and hydrated flies always showed relatively low flight activity. Visual inspection revealed that many of them were often sitting right adjacent to the water surface, over long periods of time (see Supplementary Fig. S1).

### Distribution of fly landings

**Fig. 5:**
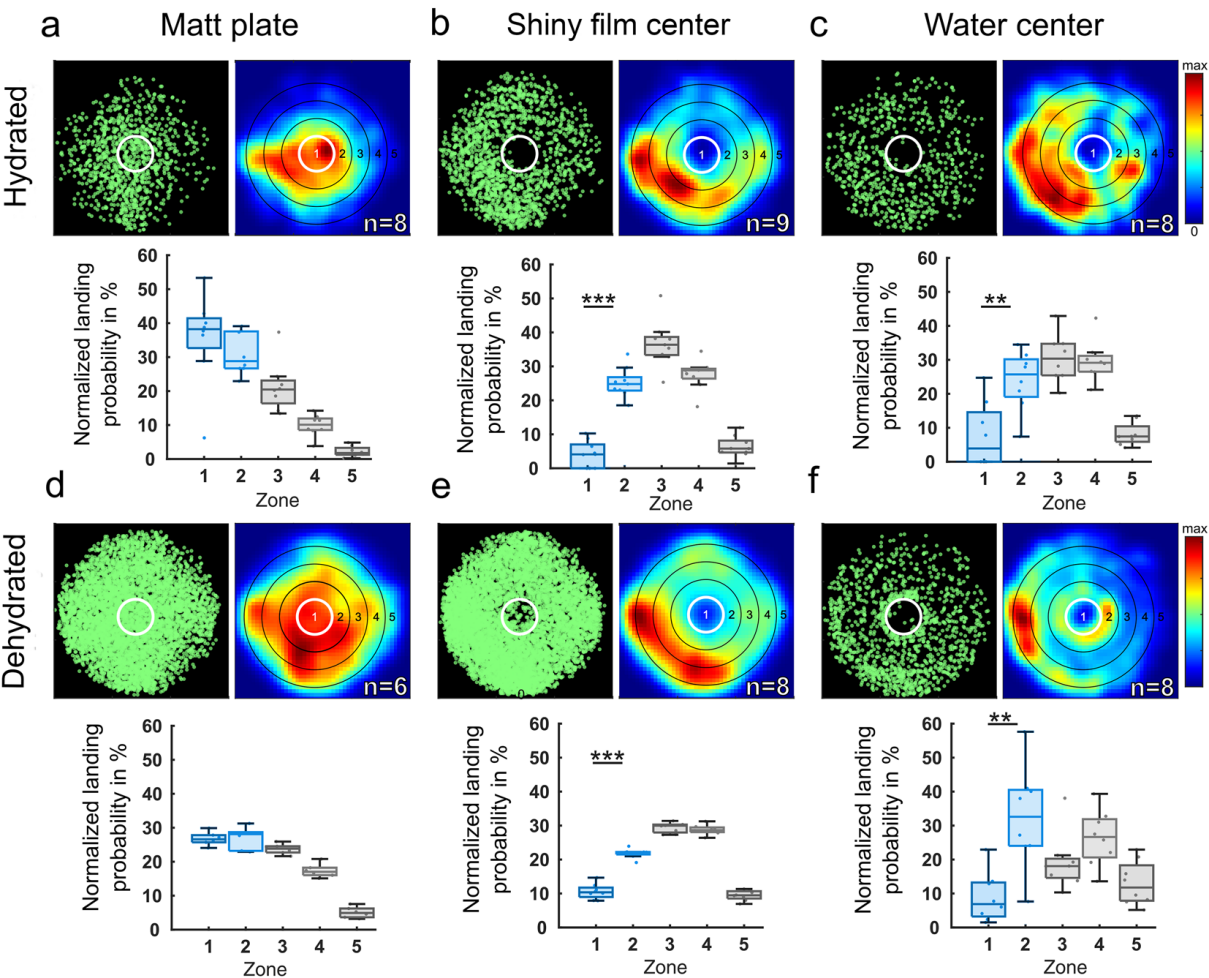
Quantification of landings of hydrated vs. dehydrated flies in response to three stimuli. **a**–**c** Summary and direct comparison of landing events of hydrated flies vs. **d**–**f** dehydrated flies in the arena after 30 min of recording of three experimental conditions: (**a**), (**d**) completely matt surface; (**b**), (**e**) matt plate with a shiny acetate film in the center; (**c**), (**f**) matt plate with a water center. For each condition, manually tracked landings are shown (green), as well as a normalized heat map of landing detections, and the normalized detection probability for landing *Drosophila* to be in each concentric zone. *n* indicates the number of 60-min recordings. *p < 0.05, **p < 0.01, ***p < 0.001

**Fig. 6:**
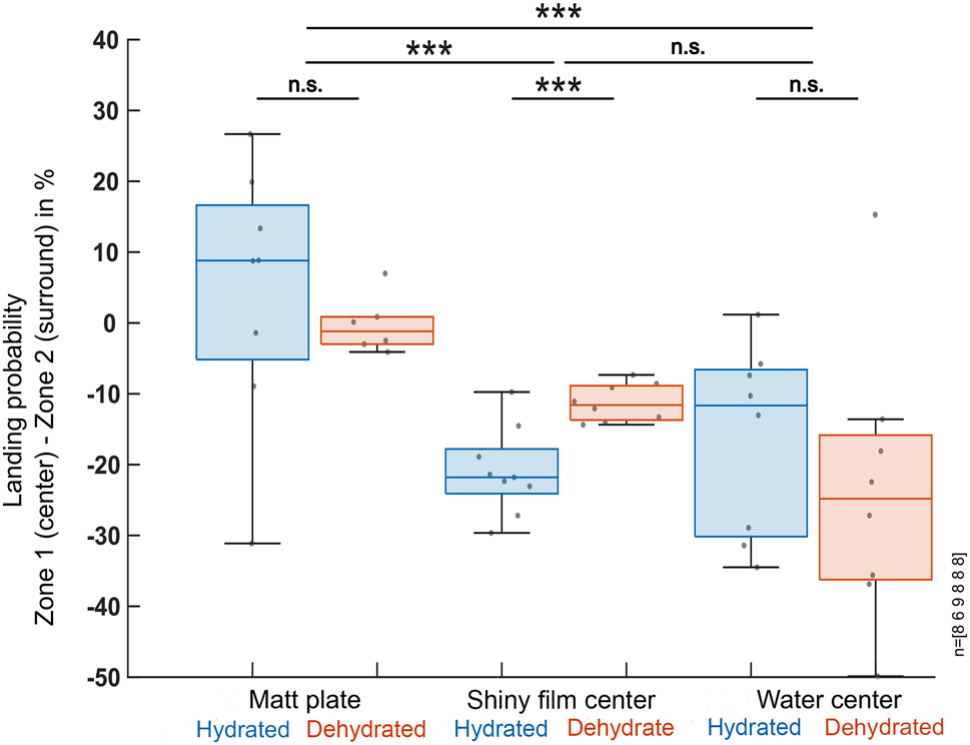
Statistical analysis of landing probabilities across all conditions. Statistical summary of landing probability (zone 1–zone 2), directly comparing hydrated and dehydrated flies in all three experimental conditions. Number of 60-min recordings from left to right indicated at the right. *p < 0.05, **p < 0.01, ***p < 0.001

Similar to our flight probability observations, the manual quantification of fly landings revealed only weak differences between landing probability in zone 1 (center) vs. zone 2 (surrounding) for the overall matt stimulus condition, both in dehydrated as well as in hydrated flies (Fig. 5a, d). In contrast, we observed a strongly reduced landing probability in zone 1 both when the shiny acetate film was placed there, as well as in the case of a real water body. This effect persisted for both dehydrated and hydrated flies (Fig. 5b, c, e, f). A direct comparison across experiments revealed that the differences in landing probability between zone 1 and zone 2 were significantly larger in those experiments using shiny acetate film or water stimuli in zone 1, as compared to the experiments where both zones 1 and 2 were matt (Fig. 6). Differences in landing probabilities for zone 1 and zone 2 were significant between dehydrated and hydrated flies only when shiny acetate film was used as a stimulus in the center. Similar to flight detection distributions, we observed a tendency of flies to predominantly land on the left side of the arena. This was likely an artifact due to a temperature gradient (< 1 °C) caused by the air conditioner in the room.

### Response to water in the darkness

**Fig. 7:**
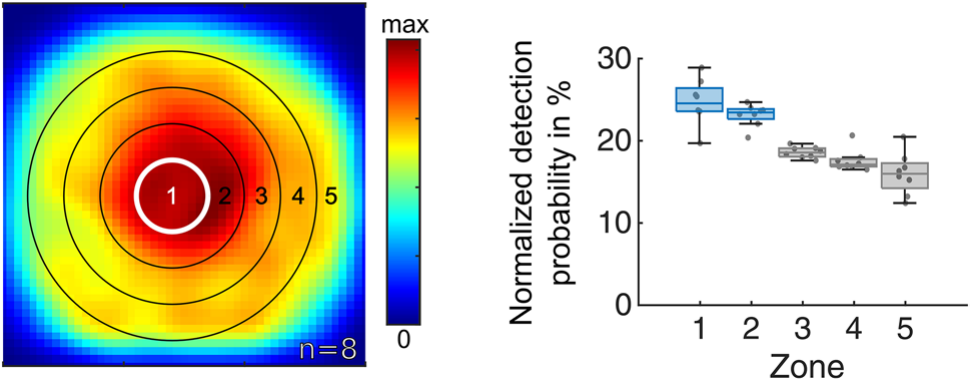
Flight densities of hydrated flies in response to water in complete darkness. Summary of flight detections of hydrated flies in the arena after 60 min of recording over a matt plate with a water center in complete darkness. A normalized heat map flight detections is shown, as well as the normalized detection probability for flying *Drosophila* to be in each concentric zone

To test whether flying flies also interact with the centrally placed water body in the complete absence of any visual cues, we also quantified the spatial distribution of flying flies in darkness with the water stimulus in the center (8 × 1 h trials, number of flies per trial: 130, 103, 104, 111, 73, 83, 84, 108, respectively). Indeed, we found that flies showed a slightly higher detection probability in zone 1 as compared to zone 2 (Fig. 7). More importantly, visual inspection of their landing behavior revealed that even without visual cues, flies would seek out the edge of the water body, where they would then remain immobile for long periods of time (Supplementary Fig S1c).

## Discussion

The experiments described here used the quantitative analysis of both flight detections and landings and revealed that flies manifest a statistically significant tendency to avoid flying over and landing on shiny surfaces, which are significantly more polarizing than the matt surroundings. Significant differences between hydrated and dehydrated flies were only found in the landing probability with a linearly polarizing center (shiny acetate film). When using water as a reflecting surface, statistical analysis did not reveal any similar effect on the spatial distribution of flight detections, neither for dehydrated nor for hydrated flies. Interestingly, most flies were found in close vicinity to the water body in complete darkness.

Sensitivity to polarized light emanating from the ventral field of view has been described for many other insect species (reviewed by Heinloth et al. 2018 and Yadav and Shein-Idelson 2021). Although the experimental setup used here is very different from the ones before, the results obtained here are in quite good agreement with these studies, as well as with those using other model systems. While most species show a strong attraction to water/shiny surfaces, only relatively few display an avoidance of such stimuli (Shashar et al. 2005).

### A new experimental setup for studying non-celestial polarization vision

The experimental setup used here represents a new, easy-to-use approach for insect behavioral science, enabling the examination of the ventral perception of linearly polarized reflections using free-flying flies and a robust algorithm for tracking flight maneuvers. Using a population of free-flying *Drosophila* allows for approximating more naturalistic behaviors, yet the setup is much easier to use than comparable 3D tracking approaches (Straw et al. 2011; Stowers et al. 2017). We chose matt acrylic surface as a negative control, since these materials are known to produce only weakly linearly polarized reflections (Kriska et al. 2007; Horvath et al. 2011). In contrast, shiny acetate film can induce behavioral responses similar to water seeking (Kriska et al. 1998; Wildermuth 1998). In this new assay, large number of free-flying insects can now be confronted with one crucial visual signal usually provided by water in nature (Kriska et al. 1998), without any interference of non-visual cues associated with water.

The bright, white arena used here reduces the amount of visual information that free-flying flies can orient to, thereby increasing the likelihood that the shiny surface will influence their behavior. Nevertheless, this visual environment remains highly unusual and is quite different from what the flies would encounter in nature. Nevertheless, we observe a tendency of the flies to avoid flying over, or land on the shiny acetate foil, which is reminiscent of the behavioral responses observed in other insect species (Mathejczyk and Wernet 2017; Heinloth et al. 2018; Yadav and Shein-Idelson 2021). Nevertheless, behavior in the wild may include components or mechanisms not tested with this assay and stimulation. For instance, water-finding behavior could be driven not just by the presence of horizontally polarized cues, but also by the presence of contrasting gradients or edges between an unpolarized land and polarized water. The landing (to drink) system then requires spatial contrast sensitivity in polarization with a behavioral filter that matches the boundary between land and water, rather than a basic positive or negative polarotactic response. Hence, future studies with similar yet improved assays are needed to deepen our understanding of the underlying mechanisms.

### Differences in detecting polarized reflections vs. water

Characterization of the water stimulus revealed very low values for the degree of polarization, almost identical to the matt negative control. Since the shiny surfaces of both water and the acetate film can produce polarized reflections, we were surprised by the relatively higher DoLP of the acetate film compared to the water stimulus. We suspect that the higher DoLP of the acetate film may be caused by the film not being glued to the white acrylic plate, essentially creating two air–water interfaces in comparison to water which has direct contact to the acrylic plate. Great care was taken to make the water and acetate film stimuli as visually similar as possible. Although the center indentation in the acrylic base plate allowed for precise and contained application of water, the surface tension of water created convex and concave surface distortions which may cause a different appearance of the stimulus edge and slightly different reflections compared to the flat acetate film (Supplementary Fig. S2).

Water had to be placed on a white surface to ensure good contrast for filming and ultimately tracking the flies. However, white surface color was previously shown to cause low polarization contrast (Meglic et al. 2019). According to these measurements, the DoLP was lowered due to the unpolarized light randomly reflected (scattered) from the white surface. Hence, the water stimulus most likely served as an unnatural stimulus, by lacking the most important visual aspects of water (reflectance and polarization), while providing all non-visual qualities of a water source, first and foremost olfactory and gustatory cues (Lin et al. 2014). Given that our behavioral paradigm allowed for the presentation of those visual cues associated with water, while omitting the olfactory and gustatory components, it seems plausible to assume that polarized reflections alone induce an overall avoidance response. Flies avoided flying over, and landing on such surfaces. However, in the absence of all visual cues, the olfactory/gustatory components also appear to be sufficient for inducing an avoidance to crash into the water body. This is in good agreement with previous studies demonstrating that flies are able to perceive water non-visually, i.e., using the gustatory sense for measuring the humidity gradient (Ji and Zhu 2015). However, to allow for better comparability between the acetate film and water, future experiments should involve a darker (gray) floor for the arena. This would reduce the amount of unpolarized light scattered from the base plate, enhancing the contribution of any reflected polarized component, thereby increasing the strength of the polarization cue.

### The effect of desiccating flies

After 6 h of desiccation, *Drosophila melanogaster*, which under normal conditions are repelled by water, become attracted to it, and this period is sufficient to induce a water-seeking behavior in *Drosophila* (Lin et al. 2014). When confronted with a shiny surface (acetate film), we found a significant difference between hydrated and dehydrated flies only in landing probabilities. We found no desiccation-based differences in flight distributions. This once again suggests that shiny surfaces mimic only the visual feature of real water, while missing crucial, non-visual features that are necessary for identifying the appetitive or aversive nature of this stimulus. The visual features alone appear to induce the same avoidance response, irrespective of the hydration of the animal. But why do desiccated flies also avoid flying over a shiny surface, if such a stimulus would signal the potential presence of water? One interpretation could be that in both conditions (hydrated and non-hydrated), flies induce similar maneuvers in response to the shiny film. For instance, flies could fly circles around the stimulus, to evaluate its relevance, or to land in close proximity to it. Such flight paths would then result in the observed decrease of flight detections right above the shiny stimulus. However, the camera tracking strategy used here is not capable of detecting such maneuvers, which is why 3D tracking solutions will be helpful in the future (Straw et al. 2011; Stowers et al. 2017). Such techniques would also allow for monitoring the tracked flies’ altitude. For instance, it is possible that from a distance, free-flying flies cannot see the relatively small water body (thus do not mind flying over it) and when they get closer to a landing, perhaps it does come into view. This would explain the discrepancy between the measurements of stimulus intensity and DoLP of the water surface used here and the behavioral results.

### The retinal substrate of ventral polarization vision

To the present day, the exact nature of the retinal substrate responsible for *Drosophila* behavioral responses to ventrally presented polarized light remains highly unclear. No ommatidia with polarization-sensitive photoreceptors reminiscent of the DRA type exist in the ventral part of the adult fly eye, i.e., two groups of photoreceptors with untwisted microvillar rhabdomeres, oriented orthogonally to each other (Wada 1974a, b; Labhart and Meyer 1999). Among flies, in horse flies, a clear difference in polarization sensitivity between pale and yellow-like ommatidial types has been found (Meglič and Wernet 2019), with the untwisted R7H / R8H photoreceptor pairs (harboring horizontally and vertically aligned microvilli in R7 and R8, respectively) being perfectly suited for polarotaxis (Stavenga et al. 2003; Meglič and Wernet 2019). Interestingly, in *Drosophila*, the same pale inner photoreceptor (R7) subtype is required to mediate ventral polarotaxis, but not its yellow R7 counterpart (Wernet et al. 2012). Hence, it remains a possibility that the underlying mechanisms are conserved among flies. In contrast, a very different organization of non-DRA ommatidia with polarization-sensitive photoreceptor subtypes was described in another fly species, the Dolichopodid fly *Sympycnus lineatus* (Trujillo-Cenóz and Bernard 1972).

Future studies should focus on revealing how polarized reflections are detected in the *Drosophila* eye. This knowledge is crucial for understanding how these signals are further processed by the brain, to inform behavioral responses. So far, the underlying pathways are understood only for the detection of celestial polarization, in some species (Weir et al. 2016; Heinze 2017; Omoto et al. 2017; Timaeus et al. 2020; Hardcastle et al. 2021; Kind et al. 2021; Sancer and Wernet 2021; Homberg et al. 2022). The behavioral setup we present here now provides an efficient platform for the systematic dissection of the retinal substrate, as well as the underlying circuit elements in *Drosophila melanogaster* (Simpson 2009; Wernet et al. 2014).

### Supplementary Information

Below is the link to the electronic supplementary material.

Supplementary file1 (TIF 261764 KB) Flight arena setup. **a**,**b** Descriptive photographs of the flight arena setup with all main components (see material & methods). **c**-**e** Photographs of the three different stimuli used here, namely a white matt plate (c), a circular shiny acetate film in the center of the white, matt plate, and (d) a circular patch of water placed in the center of a white plate (e) were taken under the experimental light conditions

Supplementary file2 (TIF 14799 KB) Flight activity over the water stimulus. These snapshots show the recording of the free-flying wild-type Drosophila melanogaster (top banana) approximately 30 minutes after being released in the arena. The flies were ventrally excited by reflected light on a water surface. By the hydrated flies (A), the thirsty flies (B), and by hydrated flies in the darkness (C). the presence of the flies around the water surface in the darkness suggests the use of additional sensory cues for water detection

Supplementary file3 (PDF 78 KB) Summary table of the flight and landings experiment. Table including the number of hydrated and dehydrated flies detected during each flight or landing experiment involving either a matt plate, a shiny center and a water center

## Data Availability

Original data is available from the authors upon request.
