# Supplementary figures and images for: Behavioral responses of free-flying Drosophila melanogaster to shiny, reflecting surfaces
Source: J Comp Physiol A Neuroethol Sens Neural Behav Physiol. 2023 Oct 5;209(6):929–41. doi: 10.1007/s00359-023-01676-0 (PMC10643280; doi:10.1007/s00359-023-01676-0)

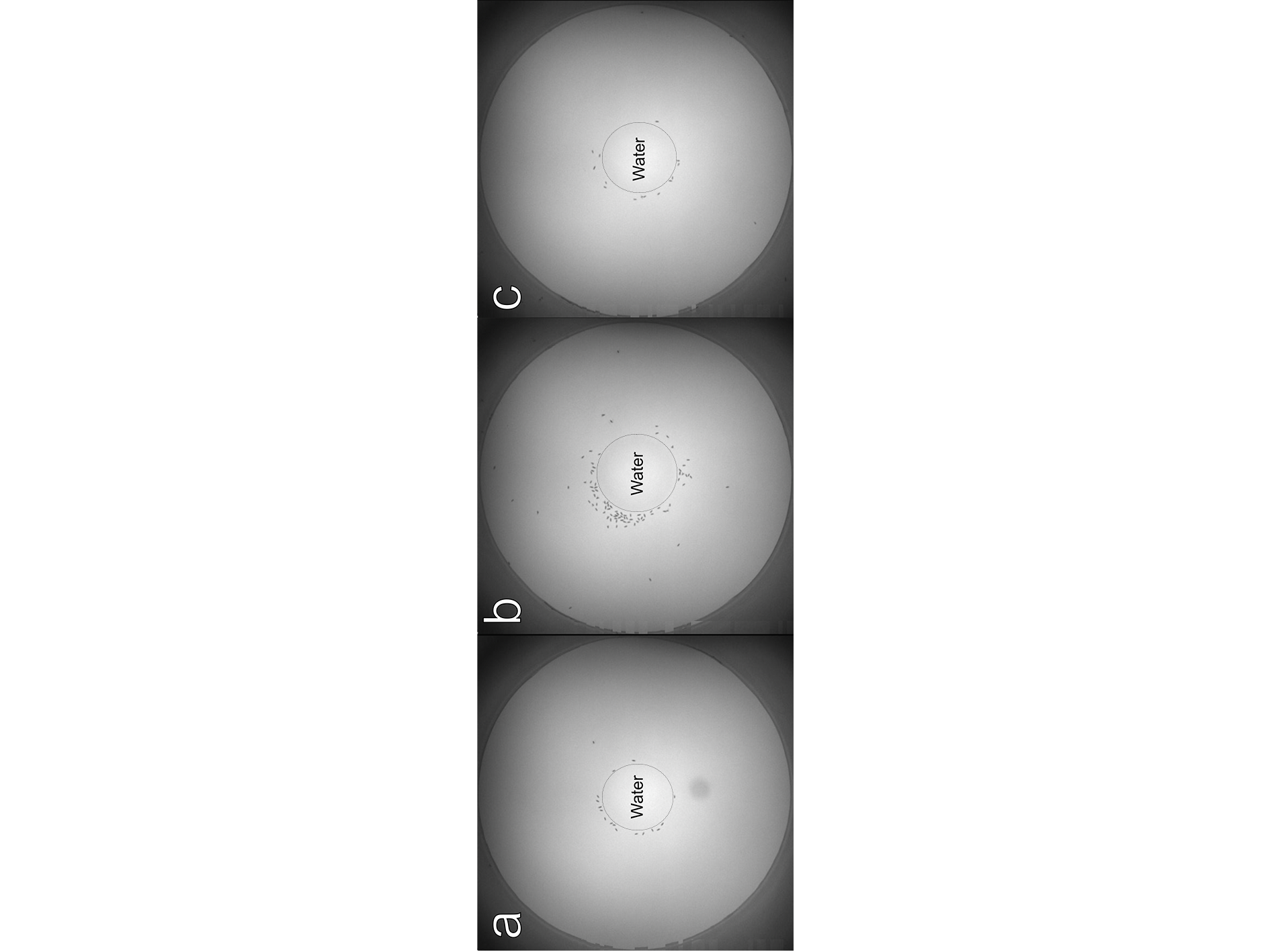

Supplement: Supplementary file 2 — Supplementary file2 (TIF 14799 KB) Flight activity over the water stimulus. These snapshots show the recording of the free-flying wild-type Drosophila melanogaster (top banana) approximately 30 minutes after being released in the arena. The flies were ventrally excited by reflected light on a water surface. By the hydrated flies (A), the thirsty flies (B), and by hydrated flies in the darkness (C). the presence of the flies around the water surface in the darkness suggests the use of additional sensory cues for water detection [file 359_2023_1676_MOESM2_ESM.tif]
