# Supplementary material for: Behavioral responses of free-flying Drosophila melanogaster to shiny, reflecting surfaces
Source: J Comp Physiol A Neuroethol Sens Neural Behav Physiol. 2023 Oct 5;209(6):929–41. doi: 10.1007/s00359-023-01676-0 (PMC10643280; doi:10.1007/s00359-023-01676-0)
Supplement: Supplementary file 3 — Supplementary file3 (PDF 78 KB) Summary table of the flight and landings experiment. Table including the number of hydrated and dehydrated flies detected during each flight or landing experiment involving either a matt plate, a shiny center and a water center [file 359_2023_1676_MOESM3_ESM.pdf]

Flight							Landings				
Hydrated			Dehydrated				Hydrated			Dehydrated	
**Number of flies**	**Number of detections**		**Number of flies**	**Number of detections**			**Number of flies**	**Number of landings**		**Number of flies**	**Number of landings**
Matt plate			Matt plate				Matt plate			Matt plate	
211	280730		122	722480			211	95		122	1000
150	257180		119	708580			150	57		108	1000
131	229850		108	929900			131	71		47	1000
98	204440		111	923090			98	101		99	1000
149	342320		106	474520			149	231		86	1000
116	146270		121	620160			116	73		110	1000
122	323610		47	238540			122	243			
207	467940		99	674580			207	160			
129	408880		86	772840							
107	603140		110	671660							
Shiny film center			Shiny film center				Shiny film center			Shiny film center	
111	211390		127	579930			111	39		127	1000
101	310170		122	563460			101	189		122	1001
78	181790		161	717170			78	230		161	1000
85	335120		179	902230			85	345		95	1000
175	501990		120	508490			175	175		115	1000
130	311580		95	456500			130	127		77	1000
105	246690		115	536580			116	85		110	1000
116	386540		77	425320			111	74		113	1001
111	335880		110	553080			114	250			
114	510610		113	485630							
Water center			Water center				Water center			Water center	
150	164810		60	80077			150	105		60	96
90	109890		120	186660			90	70		120	161
170	120410		90	175020			170	94		90	240
90	122970		20	18588			90	62		20	25
80	204750		60	48488			80	56		60	106
170	248450		20	60042			170	122		20	20
110	71571		110	221280			110	82		110	224
120	186860		110	222620			120	119		110	389
